# Short-Term Exposure to Mobile Phone Base Station Signals

**DOI:** 10.1289/ehp.10733

**Published:** 2008-02

**Authors:** Stelios A. Zinelis

**Affiliations:** Hellenic Cancer Society, Cefallonia, Greece, E-mail: zinelis@otenet.gr

The data in the study by Eltiti et al. (2007) do not support their conclusion that

The present data, along with current scientific evidence, leads to the conclusion that short-term rf-emf [radio frequency electromagnetic fields] exposure from mobile phone technology is not related to the levels of well-being or physical symptoms in IEI-EMF [idiopathic environmental intolerance with attribution to electromagnetic fields] individuals.

In the study by Eltiti et al. (2007), the intensity of the radiation emitted by the mobile phone base station was 1 μW/cm^2^ (5 mW/m^2^ for 900 MHz and 5 mW/m^2^ for 1,800 MHz). The authors assumed that the participants would not react to higher intensities such as 10 or 20 μW/cm^2^, or even to intensities up to 900 μW/cm^2^, which are used in mobile phone technology.

The exposure durations were too short to produce real effects at the biochemical and clinical levels. Ahmed et al. (2004) and Lai et al. (1992, 1994) concluded that the response depends on the duration of the radiation exposure. After 1 hr of exposure, alterations of certain biochemicals, which could be producing the symptoms, may or may not occur. For example, an increase in acetylcholinesterase activity is responsible for the levels of acetylcholine and with other neurotransmitters responsible for cognitive functions; with further exposure, this activity increases in two areas of the brain, the hippocampus and the striatum. Also, Johansson (2006) reported that electromagnetic fields may stimulate mast cells, which produce histamine, and then symptoms are produced in the skin and other organs.

Furthermore, the effects of electromagnetic fields (Belyav 2005) may be related not only to intensity or duration of exposure but also to other parameters, such as frequency or modulation.

To classify a clinical symptom as psychological, first we must exclude biochemical changes that could be triggered by the electromagnetic fields and cause neurobehavioral responses. This is supported by studies that show changes in neurotransmitters [e.g., acetylcholine (Ahmed et al. 2004), γ-aminobutyric acid (Kolomytkin et al. 1994), glutamate (Wieraszko et al. 2004)], histamine (Johansson 2006), and somatostatin (Johansson 2006)] as well as their correlation with the clinical symptoms.
